# One for all: Mating compatibility among various populations of olive fruit fly (Diptera: Tephritidae) for application of the sterile insect technique

**DOI:** 10.1371/journal.pone.0206739

**Published:** 2018-11-01

**Authors:** Sohel Ahmad, Ihsan ul Haq, Carlos Cáceres, Ulysses Sto Tomas, Thilakasiri Dammalage, Keke Gembinsky, Hannes Paulus, Marc J. B. Vreysen, Polychronis Rempoulakis

**Affiliations:** 1 Joint FAO/IAEA Division of Nuclear Techniques in Food and Agriculture, Insect Pest Control Laboratory, Vienna, Austria; 2 University of Vienna, Wien, Austria; 3 National Agricultural Research Centre, Islamabad, Pakistan; 4 Macquarie University, Department of Biological Sciences, Sydney, NSW, Australia; University of Thessaly School of Agricultural Sciences, GREECE

## Abstract

The olive fruit fly, *Bactrocera oleae* (Rossi), is the most important insect pest for the cultivation of olives worldwide. Considerable research efforts have been invested in the past decades to develop eradication or suppression tactics for use within an area-wide integrated pest management (AW-IPM) approach that includes a sterile insect technique (SIT) component. One of the major obstacles encountered in the development of SIT for olive fruit fly was the inferior quality of the mass-reared flies, expressed among others evident primarily by sterile males having a different timing of peak mating and a lower mating propensity in comparison with their wild counterparts. In this study we assessed the mating behaviour and mating compatibility of olive fruit flies originating from four countries of the Mediterranean region (Croatia, France, Italy, Spain) in walk-in field cages and post zygotic compatibility (expressed as % egg hatch) under laboratory conditions. Furthermore, we tested the hypothesis whether a hybrid strain (Greece (domesticated)/Israel (wild)) adapted to laboratory rearing conditions showed any mating barriers with all the four “wild” populations. Finally, we examined the effect of colonization on the mating compatibility of the four newly established populations over three consecutive generations. The results showed no pre-zygotic (mating barriers) or post-zygotic isolations (measured by egg hatch%) among the olive fruit fly populations from the four countries tested. Also, there was no evidence of mating barriers between the hybrid strain and the wild populations of the Mediterranean region.

## Introduction

The olive fruit fly, *Bactrocera oleae* (Rossi) (Diptera: Tephritidae), is a monophagous species that feeds exclusively on the pulp of olive fruit *Olea europaea* and some sibling species, such as *O*. *verrucosa* and *O*. *chrysophylla*. The olive fruit fly is one of the most damaging pests of olive fruit and if uncontrolled can result in more than 90% crop losses in commercial orchards [1], mostly due to fruit loss and deterioration of oil quality [2]. Traditional control measures have relied heavily on insecticide sprays [3,4], but the development of resistance of the flies against these chemicals [5], the accumulation of insecticide residues in olive fruits and oil, and the collateral damage caused by these toxic products to the environment by insecticides make them unsuitable candidates for managing this pest [6]. Furthermore, high-end prices of olive products and by-products and increased public awareness of the environmental and health impact of these insecticides were the main drivers for the growing demand for more efficient and environment friendly olive fruit fly management strategies [7,8].

The sterile insect technique (SIT) is an environment friendly control tactic that requires the rearing of insects of the target population in large numbers, sterilizing them by ionizing radiation, and releasing them in the target area where they will transfer their sterile sperm to wild females. The inseminated eggs will produce unviable embryos and as a result, there will be no offspring [9,10]. Systematic and successive releases of high quality sterile male flies in adequate over-flooding ratios can reduce the wild target population to a very low level and in certain cases eradication might be achievable [10].

During the last five decades, the SIT has been successfully incorporated in area-wide integrated pest management (AW-IPM) programs [11] against several fruit fly species, tsetse flies, New World screwworm and some lepidopteran pests in many parts of the world [12]. The SIT has been successfully applied against the Mediterranean fruit fly, *Ceratitis capitata* (Wiedemann), in several countries using prevention, suppression, containment, or eradication strategies [13,14]. In some AW-IPM programs that have an SIT component, the sterile males are produced in the vicinity of the target area, but there are also several examples of programs that transport the sterile insects from a rearing facility that is located in a different country or region. One example is the El Pino mass-rearing facility in Guatemala that produces sterile male Mediterranean fruit flies which in addition to local use, are shipped and released in southern Mexico, Florida and California, USA [15]. Another example is the Mediterranean fruit fly facility in Valencia, Spain, that has the potential to provide sterile male flies to other European regions, such as Croatia, where the SIT is in corporated in an AW-IPM programme in the Nereva River Valley to manage a local Mediterranean fruit fly population [16,17]. Production of sterile insects in one location and release in a different one can only be successful when there are no mating barriers between the sterile and the wild males and when the sterile males are competitive with the native wild males for matings with the wild females.

Because of its economic importance and the need for frequent insecticidal sprays to protect the olive crop, the olive fruit fly was among the first insects to be considered for SIT application following the New World screwworm success in the USA. Furthermore, the monophagous nature of the olive fruit fly and its dependence on the periodic phenology of olive trees (i.e. high populations are observed only during a specific time of the year that coincides with abundance of mature olive fruits) make it an ideal candidate for its management by SIT application. Despite the research and development efforts during the last four decades [7,18], the SIT has so far not been applied against this pest beyond small field experiments [7], primarily due to the lack of an efficient and cost-effective mass-rearing system. In addition, the low mating competitiveness of the reared males was another important obstacle for the implementation of the SIT [19–21]. In general, long-term rearing under artificial conditions negatively affects behavioural and physiological aspects of insects [22] and cross mating of laboratory adopted flies with wild flies has been utilized in the past for restoring quality and fitness traits of insects in mass-rearing facilities [23].

The present study was carried out to assess pre-zygotic and post-zygotic mating compatibility among olive fruit fly populations from different geographical areas and to evaluate their mating compatibility with a hybrid laboratory strain to assess its potential for use in programmes with an SIT component.

## Materials and methods

### Source of flies

Wild flies were collected in 2011 from privately owned olive orchards by the owners responding to our request to use the flies for research purposes. Samples were obtained as larvae from infested olive fruits from Croatia (Dalmatia, Latitude: 43.5089°N; Longitude: 16.4391°E), France (Nezignanl’eveque, Latitude: 43.4218°N; Longitude: 3.4066°E), Italy (Ospedaletti, Latitude: 43.8021°N; Longitude: 7.7177°E), and Spain (Valencia, Caudete de las Fuente, Latitude: 39.3330°N; Longitude: 1.1643°W), and four colonies were established at the Insect Pest Control Laboratory (IPCL) of the Joint FAO/IAEA Division of Nuclear Techniques in Food and Agriculture in Seibersdorf, Austria.

Evaluation of mating compatibility was carried out with flies from these colonies when colonised for < 5 generations in the laboratory and were therefore considered “wildish” though denoted as wild in the paper [24]. In addition, one laboratory-adapted hybrid colony was used that was developed by back-crossing wild male flies originating from a population from Israel (Beit-Dagan area, Latitude: 32.0018°N; Longitude: 34.8297°E) with females from a laboratory-adapted population (more than 300 generations) from Greece (Democritus strain). The backcrossing was conducted for four consecutive generations, resulting in ~95% estimated contribution of wild genetic material.

This study was conducted at the Insect Pest Control Laboratory of the Joint FAO/IAEA Division of Nuclear Techniques in Food and Agriculture, in Seibersdorf, Austria. Our mandate, given to use by the member States of the International Atomic Energy Agency, which is a specialised Agency under the United Nations is to develop environmental friendly control tactics for insect pests. For this we need samples from populations in these member States, as were provided by collaborators in these Mediterranean countries for the current paper. For this, we did not require authorization from regulatory bodies.

Olive fly is considered the main pest of olive production in the Mediterranean region, responsible for economic losses of several millions of Euros each year. Farmers are demanding environment friendly methods to control this pest and were therefore very eager to assist us with sending populations of these pest insects for our research. Olive fly is considered a pest, and is therefore not endangered or protected.

### Rearing of flies

Olive fruit flies were reared in small Plexiglas cages (40 ×30 ×30 cm) for the first two generations using olives or grapes [25] as an oviposition substrate to collect fertilized eggs [25]. Thereafter, the collected eggs and the young (L1) larvae were transferred on to an artificial larval medium as described in [26].

### Field cage

All mating compatibility tests were carried out in screened cylindrical walk-in field cages each containing a host tree with a height of 1.25 m and a canopy of 1.1 m in diameter. The cages had a nylon mesh screen (1 mm diameter) with a base of ca.4.0 m^2^ and a height of 1.8 m. They were placed inside a large insect “greenhouse” (20 ×10 m) with a transparent roof that provided natural daylight and that allowed temperature and relative humidity to be controlled (25 ± 1°C, 65 ± 5%). Mating tests were conducted using 10 ± 1 day-old, sexually matured male and female flies.

### Mating compatibility tests

#### General methodology

Flies used for mating compatibility tests were maintained at a density of 100–200 flies per cage (cylindrical cages 20 cm in diameter and 27 cm in height) and provided with an 8:2:0.6 mixture of sugar: enzymatic yeast hydrolysate: egg yolk and water *ad libitum* [27]. Adult flies were sexed within the first three day of emergence well before they attained sexual maturity (8–10 d). Mating tests were carried out in pairwise comparisons (involving two wild populations or one wild population and one hybrid at a time). Marking of the flies was done by dusting the pupae with fluorescent dye powder (DayGlo, US) of different colours (Stellar Green, Arch Chrome, and Orange) which is sequestered in the frontal or ptilinal suture of adults during their emergence from the puparium [28]. To avoid any bias of the colours used, they were rotated among the strains during different replications. Fifty males and fifty females from each population were released one after another at 10:30 h. Mating generally commenced between 16:00 to 16:30 h which coincided with the onset of dusk. The mating pairs were collected immediately upon detection in cotton capped 5 ml glass cylindrical vials as soon as they started mating, where they could continue to copulate. The time of the start and the end of the mating was recorded for each couple for calculating the mating duration.

### Mating compatibility test (pre-zygotic isolation)

Ten different combinations for the mating compatibility tests were used with the strains from Croatia, France, Italy, Spain, and the laboratory adapted hybrid strain (Israel/Greece), and each combination was replicated eight times. Each test used males and females from both strains resulting in four possible mating combinations, i.e. two homotypic and two heterotypic matings. The combinations (male x female) used for the tests were: France × Italy; France × Spain; France × Croatia; Spain × Croatia; Italy × Croatia; Spain × Italy; Israel/Greece × France; Israel/Greece × Italy; Israel/Greece × Spain and Israel/Greece × Croatia (Table 1).

**Table 1:** Average number of homotypic and heterotypic matings recorded in mating compatibility studies in walk-in field cages using wild *Bactrocera oleae* populations from Croatia (Cro), France (Fr), Italy (It), and Spain (Sp), and a laboratory hybrid strain from Israel/Greece (Is/Gr). The letter A indicates origin of first *B*. *oleae* strain and B indicates origin of second *B*. *oleae* strain. For the mating pairs, the first letter indicates the male insect and second letter indicates the female insect. The average numbers of replicated mating values of each combination are presented (8 replicate/combination).

Strains		Mating pairs	Mating pairs	Mating pairs	Mating pairs
A	B	AA	AB	BA	BB
It	Fr	18.1±1.7	15.5±0.9	12.7±0.6	16±1.2
It	Cro	15.3±2.1	14.6±0.9	14.6±1.3	15.3±2.4
It	Sp	16.3±1	12.6±1	13.5±1.4	12.3±1.3
Fr	Cro	15.1±1.4	14.1±1.3	12.3±1.7	15.7±1.8
Fr	Sp	14.2±1.3	11±0.9	16±1.4	14.7±1.6
Cro	Sp	10.7±1.1	8.1±0.5	10.1±0.9	7.6±1
Cro	Is/Gr	13.3±0.8	16.1±1.3	17.1±1.3	11.2±0.8
It	Is/Gr	13±1	16.8±1.6	17.8±1.4	13.3±1.8
Sp	Is/Gr	10.7±0.9	8.3±1.2	11.1±0.9	9.7±1.6
Fr	Is/Gr	14±1	16±0.8	15.7±1	16±0.8

On the test day, 50 sexually mature males and 50 sexually mature females from each strain were released at 10:30 h. In studies of mating competitiveness, the sex ratio is typically biased in favour of males (2:1), but here we used equal sex ratios as the focus was on compatibility and not competition. Each cage was visited every 20 min to observe mating pairs, which were collected in vials and checked every 15–20 min to assess mating duration. The location of mating pairs was recorded (i.e., on the tree or on the cage), and after separating, the male and female were examined under a fluorescent microscope and dye color (strain) was recorded for each.

The index of sexual isolation (ISI) was used to quantify the mating compatibility between each pairwise combination. The ISI considers the number of couples obtained for each possible combination as follows [29,30],

$$\text{ISI}=\frac{\left(\text{AA}+\text{BB}\right)-\left(\text{AB}+\text{BA}\right)}{\left(\text{AA}+\text{AB}+\text{BA}+\text{BB}\right)}$$

Where AA is the number of mating pairs of males and females from strain 1, BB is the number of mating pairs of males and females from strain 2, AB is the number of mating pairs with males of strain 1 and females of strain 2, and BA is the number of mating pairs with males of strain 2 and females of strain 1.

The male relative performance index (MRPI) was defined as:

$$\text{MRPI}=\frac{\left(\text{AA}+\text{AB}\right)-\left(\text{BB}+\text{BA}\right)}{\left(\text{AA}+\text{AB}+\text{BA}+\text{BB}\right)}$$

The female relative performance index (FRPI) was defined as:

$$\text{FRPI}=\frac{\left(\text{AA}+\text{BA}\right)-\left(\text{BB}+\text{AB}\right)}{\left(\text{AA}+\text{AB}+\text{BA}+\text{BB}\right)}$$

Both indices range from -1 (all matings achieved by olive fruit fly males or females from same population) to +1 (all matings achieved by male and female flies from different population). Both performance indexes (MRPI & FRPI) were applies for other population and combination also.

Random mating for each of ten combinations was tested with a *x*^2^ test separately. All mating indices data were analysed using Sigma-Plot 11 statistical software, and one-way ANOVA (analysis of variance) was performed to analyse the mating success.

### Postzygotic compatibility experiments in small laboratory cages

For this study, the hatch of eggs deposited by females from all possible mating combinations among the four wild colonies was examined. For each mating combination, we used 5 small cages (5 x 5 x 10 cm), each containing 5 sexually mature males and 5 sexually mature females of two different strains and which were maintained at: 25 ± 1°C, 65 ± 5% RH, 14:10 LD. Eggs were collected daily, and all collected eggs were placed in rows on moist white filter paper into Petri dishes (9 cm diameter). After 5 days, the hatched larvae were examined under a stereoscope (Leica M6) and the percentage of egg hatch assessed.

## Results

### Prezygotic mating compatibility

With the exception of the France × Croatia combination (χ^2^ = 33,90, *P* = 0.03), the mating was random in all combinations tested (using pooled data from all replicates of the mating compatibility tests): Italy × France (χ^2^ = 17.98, *P* = 0.65), Italy × Croatia (χ^2^ = 23.00, *P* = 0.34), Italy × Spain (χ^2^ = 14.04, *P* = 0.86), France x Croatia (χ^2^ = 33.90, *P* = 0.03), France x Spain (χ^2^ = 23.32, *P* = 0.327), Croatia × Spain (χ^2^ = 11.91, *P* = 0.94), Croatia × Israel/Greece (χ^2^ = 9.82, *P* = 0.98), Italy × Israel/Greece (χ^2^ = 15.32, *P* = 0.80), Spain × Israel/Greece (χ^2^ = 13.74, *P* = 0.88), France × Israel/Greece (χ^2^ = 11.32, *P* = 0.95).

The ISI values also indicated random mating between the populations in all mating combinations as the 95% confidence limits were close to zero (Table 2). The same tendency of mating propensity was also followed by the MRPI and FRPI indices (Table 2). Overall, results of the mating compatibility tests indicated the complete absence of mating barriers amongst the tested wild populations and with the laboratory-adapted hybrid population.

**Table 2:** The ISI, MRPI, and FRPI indices as obtained during the field cage tests among wild olive fruit fly populations from Croatia, France, Italy, Spain, and the hybrid Israel/Greece strain.

Combination	ISI Mean (95% CL)	MRPI Mean (95% CL)	FRPI Mean (95% CL)
Italy× France	0.09(-0.03±0.14)	0.07(-0.02±0.17)	-0.01(-0.14±0.12)
Italy× Croatia	0.01(-0.07±0.09)	0.001(-0.11±0.11)	0.006(-0.13±0.12)
Italy× Spain	0.05(-0.02±0.13)	0.06(-0.04±0.17)	0.08(0.008±0.16)
France× Croatia	0.07(-0.001±0.14)	0.03(-0.14±0.22)	0.04(-0.21±0.12)
France× Spain	0.03(-0.01±0.08)	-0.09(-0.21±0.01)	0.07(-0.08±0.23)
Croatia× Spain	-0.005(-0.11±0.09)	0.033(-0.03±0.09)	0.13(0.008±0.26)
Croatia× Israel/Greece	-0.14(-0.22±0.05)	0.01(-0.03±0.07)	0.05(-0.02±0.13)
Italy× Israel/Greece	-0.13(-0.19±0.08)	0.01(-0.10±0.07)	0.02(-0.08±0.14)
Spain× Israel/Greece	0.02(-0.05±0.11)	-0.04(-0.11±0.01)	0.12(-0.03±0.28)
France× Israel/Greece	-0.03(-0.09±0.03)	-0.02(-0.12±0.07)	-0.03(-0.09±0.02)

All the replicated (n = 8) combination values were evaluated in 95% CL.

There were no statistical differences in latency to mating in any of the mating combinations: Croatia × Spain (F_3,2_ = 1.2; *P* = 0.3), Italy × Croatia (F_3,4_ = 0.7; *P* = 0.5), Italy × Spain (F_3,4_ = 0.1; *P* = 0.9), France × Spain (F_3,4_ = 0.04; *P* = 0.9), France × Croatia (F_3,4_ = 1.8; *P* = 0.1), Italy × France (F_3,4_ = 1.5; *P* = 0.1), Israel/Greece × Croatia (F_3,4_ = 0.8; *P* = 0.4), Israel/Greece × Spain(F_3,3_ = 0.3; *P* = 0.8), Italy × Israel/Greece (F_3,4_ = 0.1; *P* = 0.9) and Italy × France (F_3,4_ = 0.9; *P* = 0.4) (Table 3).

**Table 3:** Latency to mating pair of the couples [mean ± SE] in the walk-in field cage for heterotypic and homotypic crosses of five different populations of *Bactrocera oleae*.

Populations	Mating combination (Male-Female)	Latency (Minutes)
Croatia × Spain	Croatia × Croatia	460.9±6.3
	Croatia × Spain	442±6.2
	Spain × Croatia	451.0±8.8
	Spain × Spain	447.2±7.3
Italy × Croatia	Italy × Italy	419.8±4.2
	Italy× Croatia	411.4±3.7
	Croatia × Italy	418.4±4.3
	Croatia × Croatia	415.8±4.2
Italy × Spain	Italy × Italy	413.4±5.4
	Italy × Spain	416.7±6.7
	Spain × Italy	415.9±6.0
	Spain × Spain	418.1±5.9
France × Spain	France × France	420.0±4.4
	France × Spain	420.0±5.3
	Spain × France	422.1±4.5
	Spain × Spain	421.3±4.4
France × Croatia	France × France	395.6±5.1
	France × Croatia	405.9±5.3
	Croatia × France	404.3±5.2
	Croatia × Croatia	412.1±4.9
Italy × France	Italy × Italy	402.3±4.9
	Italy × France	394.7±5.0
	France × Italy	397.3±5.4
	France × France	387.0±5.5
Israel/Greece × Croatia	Croatia × Croatia	295.2±6.7
	Croatia × Israel/Greece	293.9±5.3
	Israel/Greece × Croatia	302.5±4.6
	Israel/Greece × Israel/Greece	305.6±6.8
Israel/Greece × Spain	Spain × Spain	417.3±8.1
	Spain × Israel/Greece	415.1±7.4
	Israel/Greece × Spain	416.2±10.0
	Israel/Greece × Israel/Greece	407.0±8.0
Israel/Greece × Italy	Italy × Italy	310.4±7.3
	Italy × Israel/Greece	304.9±5.7
	Israel/Greece × Italy	309.3±6.3
	Israel/Greece × Israel/Greece	307.4±7.6
Israel/Greece × France	France × France	277.1±3.5
	France × Israel/Greece	271.1±3.1
	Israel/Greece × France	273.9±3.2
	Israel/Greece × Israel/Greece	269.5±3.6

### Post zygotic mating

Except for the France × Croatia cross (F_3,1_ = 3.8; P = 0.03), there was no significant difference in egg hatch for the different crosses: Italy × France (F_3,1_ = 1.1; P = 0.3), for Italy × Spain (F_3,1_ = 0.7; P = 0.5), for France × Spain (F-_3,1_ = 0.8;P = 0.5), for Italy × Croatia (F_3,1_ = 26.5;P = 1.8) for Spain × Croatia (F_3,1_ = 4.7;P = 0.01), and for France × Croatia (Table 4). The combination of Italy and Spain population showed a comparatively better egg hatch (%) as compared with the Croatia and France populations.

**Table 4:** Fertility (% egg hatch) (mean±SE), for all possible mating combinations among four wild *Bactrocera oleae* populations originating from different geographical regions.

Populations	Mating combination (Male-Female)	Egg hatch (%)
Italy × France	Italy × Italy	89.1±3.1
	Italy × France	96.6±1.0
	France × Italy	91.5±1.1
	France x France	95.4±0.6
Italy × Spain	Italy × Italy	87.2±2.1
	Italy × Spain	85.1±3.5
	Spain × Italy	89.8±2.4
	Spain × Spain	81.3±4.8
France × Spain	France × France	68.7±7.7
	France× Spain	76.3±7.3
	Spain × France	72.9±5.2
	Spain × Spain	82.0±5.5
Italy × Croatia	Italy × Italy	82.4±4.5
	Italy × Croatia	76.1±6.7
	Croatia × Italy	86.1±3.3
	Croatia × Croatia	83.2±2.7
Spain × Croatia	Spain × Spain	87.8±1.9
	Spain × Croatia	73.9±3.7
	Croatia × Spain	82.7±1.1
	Croatia × Croatia	56.2±3.1
France × Croatia	France × France	92.5±1.9
	France × Croatia	74.8±5.8
	Croatia × France	89.7±3.8
	Croatia × Croatia	90.1±1.6

## Discussion

Insects undergo a behaviour-altering chain of processes during mass-rearing in an artificial environment and during handling and irradiation procedures before being released in the target area. The importance of mating compatibility between the released strain and the wild target population cannot be overemphasised and was already assessed with some fruit fly [31], tsetse fly [32] and Lepidoptera species [33,34]. Studies with the Mediterranean fruit fly *C*. *capitata* [35,36], and the codling moth *Cydia pomonella* [33] indicated the complete absence of mating barriers between populations from around the world. Conversely, mating isolation was clearly evident between populations of the South American fruit fly *Anastrepha fraterculus* that originated from different Latin American countries [37,38]. Mating studies have even been instrumental in the confirmation of the taxonomic status of some *Bactrocera* species that 288resulted in the synonymization of four species within the *Bactrocera dorsalis* complex (*B*. *dorsalis*, *B*. *invadens*, *B*. *philippinensis*, and *B*. *papaya*) [39].

The establishment of four new “wild” colonies of olive fruit fly from different geographic areas from the Mediterranean region offered an opportunity to assess mating compatibility amongst them. In addition, the hybrid strain (Israel/Greece) was included in these studies. The study found no evidence of behavioural mating isolation between the five tested populations of olive fruit flies with the exception of the France-Croatia combination. This suggested partial incompatibility among the France and Croatia populations, but this was however not supported by the other calculated indices. In the laboratory experiments, homotypic and heterotypic crosses displayed similar levels of fertility as assessed by egg hatch. These results corroborate the findings of the mating trials and support the absence of mating barriers as observed in the prezygotic studies.

Mating latency is considered critical for the success of the SIT as earlier matings of wild males would potentially leave less opportunity for sterile males to transfer their sterile sperm to the native female population. In this study we use the mating latency as an indicator of potential isolation, at the same time being cautious not to overestimate the importance of those findings for predicting the overall success of the tested strains. Mating latency was similar among the tested olive fruit fly populations from different geographical origins, and there was no clear evidence of spatial partition of mating location in view that most of the mating occurred on the cage walls. This finding needs to be treated cautiously, as most olive trees in the field are much larger, but on the other hand direct mating observations within tree canopies under field conditions are almost impossible. Our observations are particularly encouraging for the application of the SIT, as olive fruit fly is a dusk mating fly and the mating occurs during a well-defined time of the day. However, we consider using in future studies the exact time of mating and circular statistics as a potentially more reliable method to analyse mating behaviour of dusk mating species of fruit flies. The phenomenon of allochronic mating isolation has also been observed in nature in other fly species like the two-sibling species *B*. *tryoni* and *B*. *neohumeralis* [40]. This mechanism might act as a speciation force in sympatric populations, leading eventually to new species. With respect to the SIT, the same phenomenon might surely jeopardize the efficiency of the release component, and in the case of bisexual strains, result in assortative mating between released and wild flies respectively.

Laboratory colonization has been reported to adversely affect the quality of reared flies and one of the strategies used to reduce the quality problems is by periodic colony refreshment using backcrosses between laboratory strains and wild strain. This strategy is being practiced in many insect mass-rearing facilities [23]. Hybridizing the wild males with laboratory-adopted female insects has in many instances increased egg production of the newly developed hybrid strain, which is a benefit when using the strain in an SIT programme. A similar strategy was used at the IPCL with the olive fruit fly by using consecutive back crossings between female flies from an old laboratory strain from Greece (Democritus strain) and wild males originating from Israel. The developed hybrid strain produced significantly more eggs, and was consequently colonized and used in a pilot SIT study in Israel [7].

In view of the cumbersome protocol for oviposition and egg collection from wild strains, the hybridization approach was deemed less challenging as compared with the establishment of colonies from pure wild samples, and therefore, a hybridization process is recommended for the establishment of new olive fruit fly colonies [25]. This approach would also have the advantage of “hybrid vigor’ [25]. The olive fruit fly hybrid colony that was developed at the IPCL enabled more effective rearing of the flies without any evidence of mating barriers with all types of wild strains tested.

All data combined demonstrate that, from a qualitative point of view, olive fruit fly populations from the Mediterranean region tested have not yet evolved diverging mating behaviours indicative of incipient pre-mating isolation mechanisms affected by local natural selection. Wild populations from different regions within the Mediterranean basin were sexually compatible with each other and with a hybrid laboratory strain. The findings of the present experiments provide important information for the development of the SIT in an AW-IPM program against olive fruit fly and support the potential use and release of one strain anywhere in the Mediterranean region and possibly in the world. An important population that was not tested for compatibility in this study is the Californian olive fly population that is now established in the USA since 1998 [41]. One of the future studies will need to confirm mating compatibility involving this and possibly other introduced populations.
